# Isolation of feline islets of Langerhans by selective osmotic shock produces glucose responsive islets

**DOI:** 10.3389/fvets.2024.1365611

**Published:** 2024-03-07

**Authors:** Lauren T. Porter, Christopher A. Adin, Chiquitha D. Crews, Jocelyn Mott, Chen Gilor

**Affiliations:** ^1^Department of Small Animal Clinical Sciences, College of Veterinary Medicine, University of Florida, Gainesville, FL, United States; ^2^College of Veterinary Medicine Dean’s Office, University of Florida, Gainesville, FL, United States

**Keywords:** diabetes mellitus, islet isolation, feline, GLUT2, selective osmotic shock, β-cell

## Abstract

**Introduction:**

Pancreatic islet isolation is essential for studying islet physiology, pathology, and transplantation, and feline islets could be an important model for human type II diabetes mellitus (T2D). Traditional isolation methods utilizing collagenases inflict damage and, in cats, may contribute to the difficulty in generating functional islets, as demonstrated by glucose-stimulated insulin secretion (GSIS). GLUT2 expression in β cells may allow for adaptation to hyperosmolar glucose solutions while exocrine tissue is selectively disrupted.

**Methods:**

Here we developed a protocol for selective osmotic shock (SOS) for feline islet isolation and evaluated the effect of different hyperosmolar glucose concentrations (300 mmol/L and 600 mmol/L) and incubation times (20 min and 40 min) on purity, morphology, yield, and GSIS.

**Results:**

Across protocol treatments, islet yield was moderate and morphology excellent. The treatment of 600 mmol/L glucose solution with 20 min incubation resulted in the highest stimulation index by GSIS.

**Discussion:**

Glucose responsiveness was demonstrated, permitting future *in vitro* studies. This research opens avenues for understanding feline islet function and transplantation possibilities and enables an additional islet model for T2D.

## Introduction

In contrast to current rodent models, domestic cats naturally develop a form of diabetes mellitus (DM) that shares many similarities to type 2 diabetes (T2D) in humans, including obesity-induced insulin resistance and dyslipidemia, alterations in gut microbiota, impaired β-cell function, and spontaneous pancreatic amyloid deposition (1, 2). However, the utility of cats as a research model of type 2 diabetes has been hampered by the difficulty in studying feline islets *in vitro*. The ability to study islets *in vitro* is a critical component in the advancement of research into the prevention, targeted treatment, and etiology of DM.

The incidence of DM in cats is reported as approximately 1 in 100 cats presented to veterinary teaching hospitals (3) and approximately 1 in 200 cats presented to veterinary private practice (4). Like T2D in people, obesity, physical inactivity, and increased age are risk factors for DM in cats (5–7). Feline DM does not typically involve immune-mediated destruction of beta cells, but beta cell loss and dysfunction do occur (8–10), and unfortunately most cats are insulin-dependent upon diagnosis (11). Even with excellent owner compliance, daily insulin injections can be challenging, and DM can lead to a variety of complications such as infection, polyneuropathy, nephropathy, hypoglycemia, and diabetic ketoacidosis (8, 11). Therefore, islet transplantation might be worth exploring as a treatment option in feline DM. Whole pancreas transplantation allows for precise glycemic control but necessitates lifelong immunosuppression and is accompanied by high risk of organ rejection and perioperative complications (12–14). In contrast, islet isolation from exocrine pancreatic tissue enables targeted transplantation of only functionally necessary cells while minimizing host immune response and avoiding a surgical procedure. The site of clinical islet transplantation in people is the portal vein accessed via interventional radiology (15), but infusion into the peritoneal cavity and omentum have been reported in dogs, and may be more realistic transplant sites for the veterinary patient (16, 17). Additionally, isolated islets can be encapsulated, offering protection from the host immune system, eliminating the need for immunosuppression (15, 18). In humans, islet transplantation can result in 100% insulin independence at 1 year, and while this decreases to 30% at 5 years, recipients continue to have a 90% decrease in dangerous hypoglycemic episodes (15).

Protocols for islet isolation and culture have been developed and are routinely performed in other species, but these traditional methods involve enzymatic digestion and mechanical cell separation via density gradient centrifugation (19, 20). These methods can result in low islet yield and high rates of cell death (21–23). Consequently, most diabetic patients require multiple islet transplants to achieve satisfactory results (15). Collagenases are intended to disrupt connective tissue, but can also penetrate islet cell membranes, resulting in apoptosis, necrosis, and release of pro-inflammatory cytokines and free radicals, contributing to graft failure (24, 25). Atwater et al. (26) proposed an alternative method of islet isolation to circumvent some of these negative outcomes. This method exploits the GLUT 2 transporter to selectively spare beta cells from rapid changes in osmolality mediated by glucose concentration. In a high glucose hyperosmolar solution, glucose enters beta cells freely, preventing an osmolar gradient. Meanwhile, cells that do not express this membranal transporter are subjected to rapid changes in tonicity, cell membrane damage, and subsequent cell death. This selective osmotic shock technique has been successfully applied for islet isolation from porcine, canine, and human pancreata (26–28). Moreover, it is technically simpler and yields higher quantities compared to enzymatic digestions (26, 27).

Feline islet isolation has been described in a small number of studies with limited success utilizing traditional collagenase methods (29–32). To our knowledge, there is only one publication demonstrating yield of functional feline islets as determined by glucose-stimulated insulin secretion (GSIS) testing (32), which is the gold standard for assessing islet function (33). We hypothesized that use of selective osmotic shock (SOS) for isolation of feline islets will produce glucose-responsive beta cells. Our objectives in the current study were to (1) develop a selective osmotic shock protocol for feline islet isolation (2) describe purity and morphology and (3) compare the effects of osmolality and incubation time on islet yield and glucose responsiveness.

## Methods

### Cats

**Figure 1:**
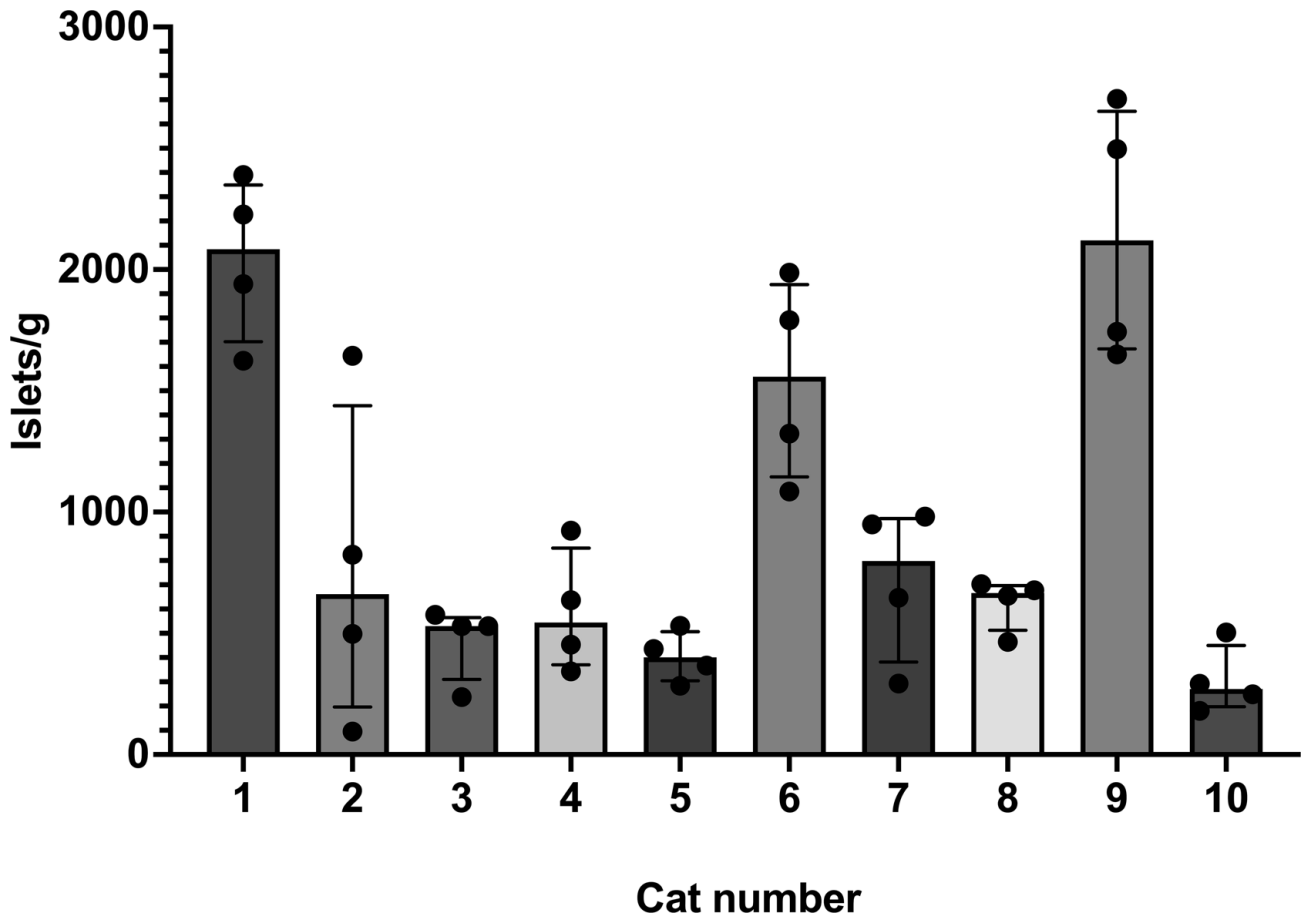
Islet yield among individual cats. Histogram (median) and interquartile range (25 and 75 percentiles) depicting islet yield for each cat, #1–10. For each cat, *n* = 4 (treatments A–D). Yield was compared between treatment groups and cats using a non-parametric ANOVA (Friedman’s) test (two tailed) and was found to be different among individual cats (*p* = 0.0025).

The study was approved by the Institutional Animal Care and Use Committee (protocol #202011101) at the University of Florida and conducted in accordance with all applicable regulations and guidelines, including the ARRIVE guidelines (34). Nine adult, aged 6–7 years, domestic shorthair cats from a laboratory colony housed at the University of Florida were used in this study. Body weight ranged from 3.9 to 7.2 kg (mean 5.3, SD 0.91). Four cats were female spayed, and five cats were male neutered. All animals were part of another study that involved partial pancreatectomy of the left pancreatic limb under general anesthesia. Anesthesia protocols were determined by the Animal Care and Use Veterinarian and consisted of premedication using Ketamine 2.5 mcg/kg, Dexmedetomidine 10 mcg/kg, and Butorphanol 0.2 mg/kg intramuscularly. Once sedated, cats underwent endotracheal intubation. Inhaled Isoflurane was administered if needed to facilitate intubation. Cats were maintained under anesthesia with inhaled Isoflurane in 100% oxygen. The resected tissue would have otherwise been discarded but instead was used in our study for islet isolation. Additional pancreatic tissue was obtained from a single cat that was euthanized for reasons unrelated to this study in accordance with AVMA guidelines for the Euthanasia of Animals (2020). This was a 2-year-old purpose-bred, male cat, and is cat 2 in Figure 1. All cats used in this study were healthy and without known pancreatic disease.

### SOS-based method of islet isolation

**Figure 2:**
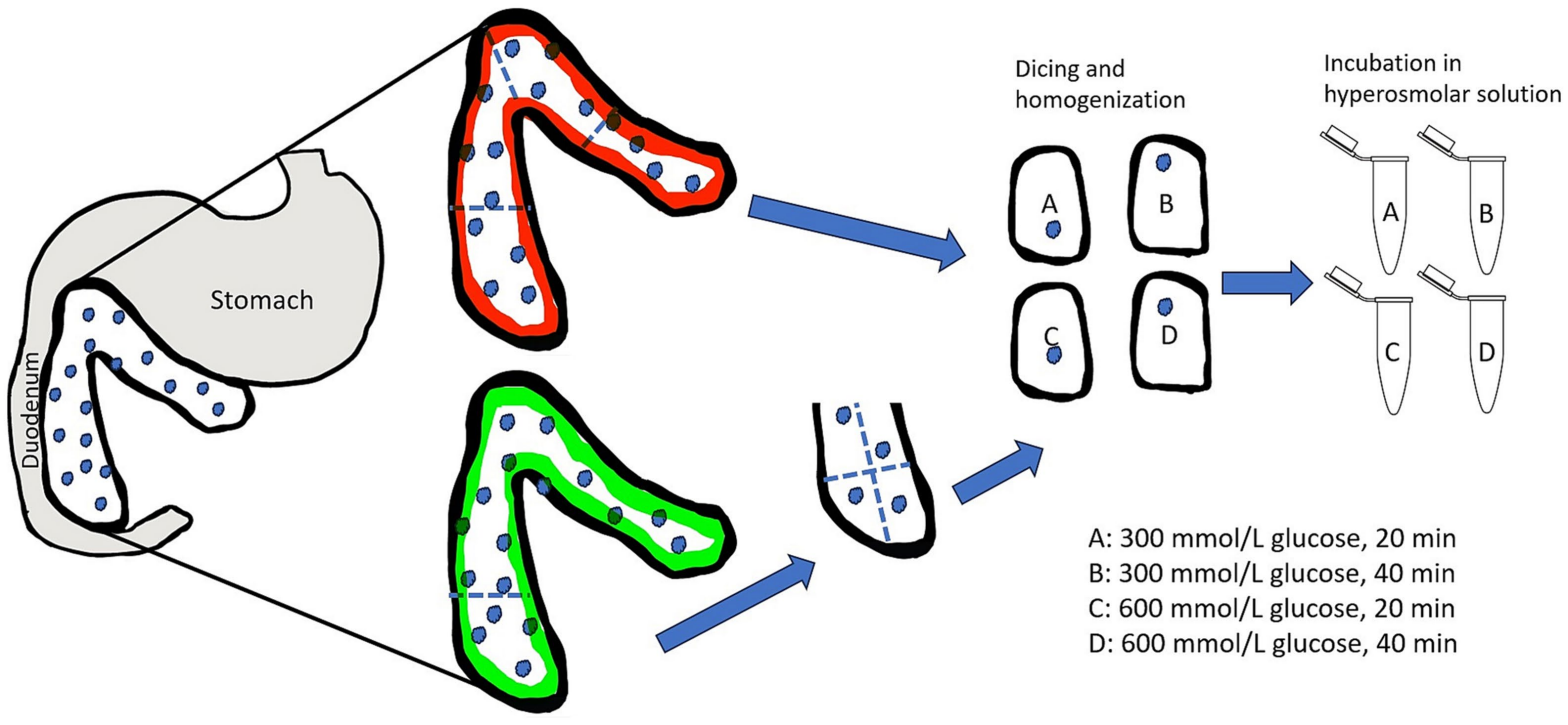
Illustration of the division of the feline pancreas and randomized treatment group assignment for selective osmotic shock. Partial pancreatectomy is represented by the pancreas outlined in green while total pancreas extraction is represented by the pancreas outlined in red. The assignment of treatment groups A–D was random for each cat to avoid bias due to asymmetrical distribution of islets. Following treatment group assignment, the tissue was mechanically disrupted and then subjected to either 300 mmol/L glucose solution or 600 mmol/L glucose solution for either 20 min or 40 min. Illustration is not to scale.

Following tissue harvest, the pancreatic tissue was submerged in RPMI 1640 media within a sterile specimen cup and covered in at least 1 inch of ice during transportation to the laboratory. Isolation was started within 15 min of harvest. Aseptic technique was used throughout the isolation method. The tissue in entirety was weighed using a gram scale and then divided into 4 comparable-sized pieces. These 4 pieces were randomly assigned to treatments A through D (Figure 2). Each segment was weighed individually using a gram scale so that yield could be standardized and reported per gram of tissue. Under a laminar flow hood at room temperature, the tissue segment was diced using a #10 surgical blade for approximately 10 min, or until fragments were < 3 mm. Each diced tissue segment was placed into 50 mL sterile centrifuge tubes with 30 mL Roswell Park Memorial Institute (RPMI) 1,640 zero glucose (Gibco™). Tissue homogenization was performed for approximately 30 s using a handheld tissue homogenizer (Tissue Tearor™, Biospec Products, Inc. Bartlesville, OK). The tubes were then centrifuged for 5 min at 180 relative centrifugal force (rcf) to produce a pellet. Media was decanted, and each homogenized tissue segment was resuspended in 30 mL of a hyperosmolar glucose solutions with the following protocols: treatment A; 300 mmol/L glucose for 20 min exposure time, treatment B; 300 mmol/L glucose for 40 min exposure time, treatment C; 600 mmol/L glucose for 20 min exposure time, and treatment D; 600 mmol/L glucose for 40 min exposure time. Experimental media were prepared prior to the experiments by adding appropriate amounts of glucose (Dextrose 50%, VetOne, Boise, ID) to glucose-free RPMI 1640 media.

The tissue fragments were periodically agitated during incubation at room temperature. After the respective treatment times, the tubes were centrifuged for 5 min at 180 rcf, media was decanted, and the pellet was resuspended in glucose-free RPMI media. Each sample was vigorously mixed and re-centrifuged for an additional 2 rinses using glucose-free RPMI for a total of 3 rinses. The tissue was then plated into 100 mm sterile polystyrene petri dishes and 3 mL of standard islet culture media composed of RPMI 1640 + 10% fetal bovine serum (Gibco™) + 1% Penicillin Streptomycin (Gibco™) and incubated at 37°C, 5% CO2. At 24 h after islet isolation, islets were hand-picked using a micropipette to increase purity and quantified per plate using an inverted light microscope (Olympus CKX53). Light microscopy imaging was performed with SPOT Idea CMOS microscope camera and SPOT Software 5.2 imaging software. Islet yield was then calculated by dividing the number of islets per treatment plate by the respective weight of the tissue segment. Purity (% islet tissue vs. % exocrine tissue) was estimated visually under light microscopy.

### Glucose-stimulated insulin secretion

Islet β-cell function was measured with a modified version of a glucose-stimulated insulin secretion (GSIS) assay used in human islet transplant centers (Integrated Islet Distribution Program City of Hope, Standard Operating Procedure for GSIS).^1^ GSIS was performed 24 h after islet isolation on pancreata from cat 3–10 (*n* = 8) and was not performed on pancreata from cats 1 and 2 due to the lack of available consumables. Approximately 20 islets of similar size were handpicked with a micropipette from each treatment group and divided between 2 Eppendorf tubes (1.5 mL), each tube containing 500 μL of glucose-free RPMI 1640. The tubes were then pre-incubated for 2 h at 37°C. After incubation, the Eppendorf tubes were spun at low speed (180 rcf, 2 min), the glucose-free media was decanted, and each treatment group had 500 μL of a low glucose concentration RPMI (2.8 mmol/L) added to one tube and a high glucose concentration RPMI (28 mmol/L) added to the other tube. The researcher performing the experiment was blinded to the assignment of high (28 mmol/L) glucose concentration and low (2.8 mmol/L) glucose concentration during the islet handpicking as to minimize bias based on islet size and morphology. After 1 h incubation at 37°C, the tubes were again spun, and the media was removed and stored in a −20°C freezer for insulin determination. Insulin was measured using a commercially available feline insulin ELISA kit (Mercodia). Stimulation index was calculated using the standard formula:


$$\begin{array}{l} \mathrm{Stimulation}\;\mathrm{Index}\;\left(SI\right)=\\ \frac{\mathrm{Insulin}\;\mathrm{concentration}\;\mathrm{after}\;\mathrm{high}\;\mathrm{glucose}\;\mathrm{stimulation}}{\mathrm{Insulin}\;\mathrm{concentration}\;\mathrm{after}\;\mathrm{low}\;\mathrm{glucose}\;\mathrm{stimulation}} \end{array}$$

**Figure 3:**
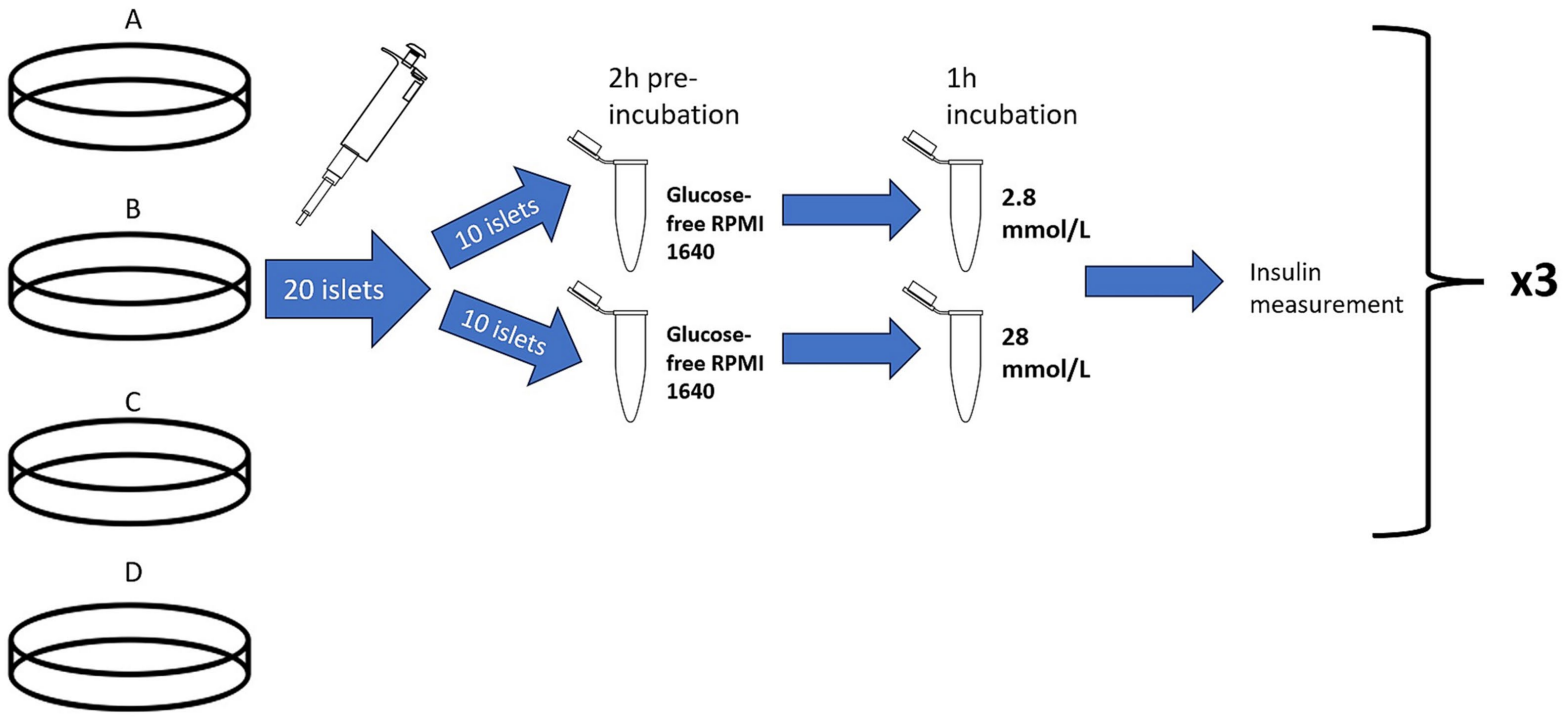
Illustration of the glucose stimulated insulin secretion test. Treatment group B, represented by cell culture plate B, is used as an example to depict the procedure used for all treatment groups (A–D). For each treatment group, represented by cell culture plate A–D, 20 islets were hand-picked with a pipette and divided into two Eppendorf tubes for a 2-h pre-incubation in glucose-free RPMI 1640. Then, one group of 10 islets was exposed to a low glucose concentration (2.8 mmol/L) and the other group of islets was exposed to a high glucose concentration (28 mmol/L). After 1 h, the supernatant from each group was saved and analyzed for insulin concentration to determine stimulation index. This was performed in triplicate for each treatment group. Illustration is not to scale.

The stimulation assay was performed in triplicates, for a total of 24 samples (approximately 60 islets per treatment group). In summary, three 10-islet Eppendorf tubes were tested at a low-glucose concentration and three 10-islet Eppendorf tubes were tested at a high-glucose concentration for each of the 4 treatment groups (treatment A–D) (Figure 3). Missing GSIS data for two pancreata in treatments B–D (*n* = 6 for these treatment groups) was due to limited sample and feline insulin ELISA kit.

### Statistics

Data were analyzed using a commercial software package (GraphPad Prism, Version 10.0.2). Because of the small sample size, all data were analyzed with nonparametric tests and reported as median and range. For GSIS data, triplicates were first averaged into a single data point per treatment. Yield was compared between cats and between treatments using non-parametric ANOVA (Friedman’s) test (two-tailed). For GSIS, missing data precluded a repeated-measure analysis and treatments were compared using the Kruskal-Wallis test followed by Dunn’s test for multiple comparisons with adjusted *p*-values (two-tailed). Significance level was set to 0.05.

## Results

### Islet yield

**Figure 4:**
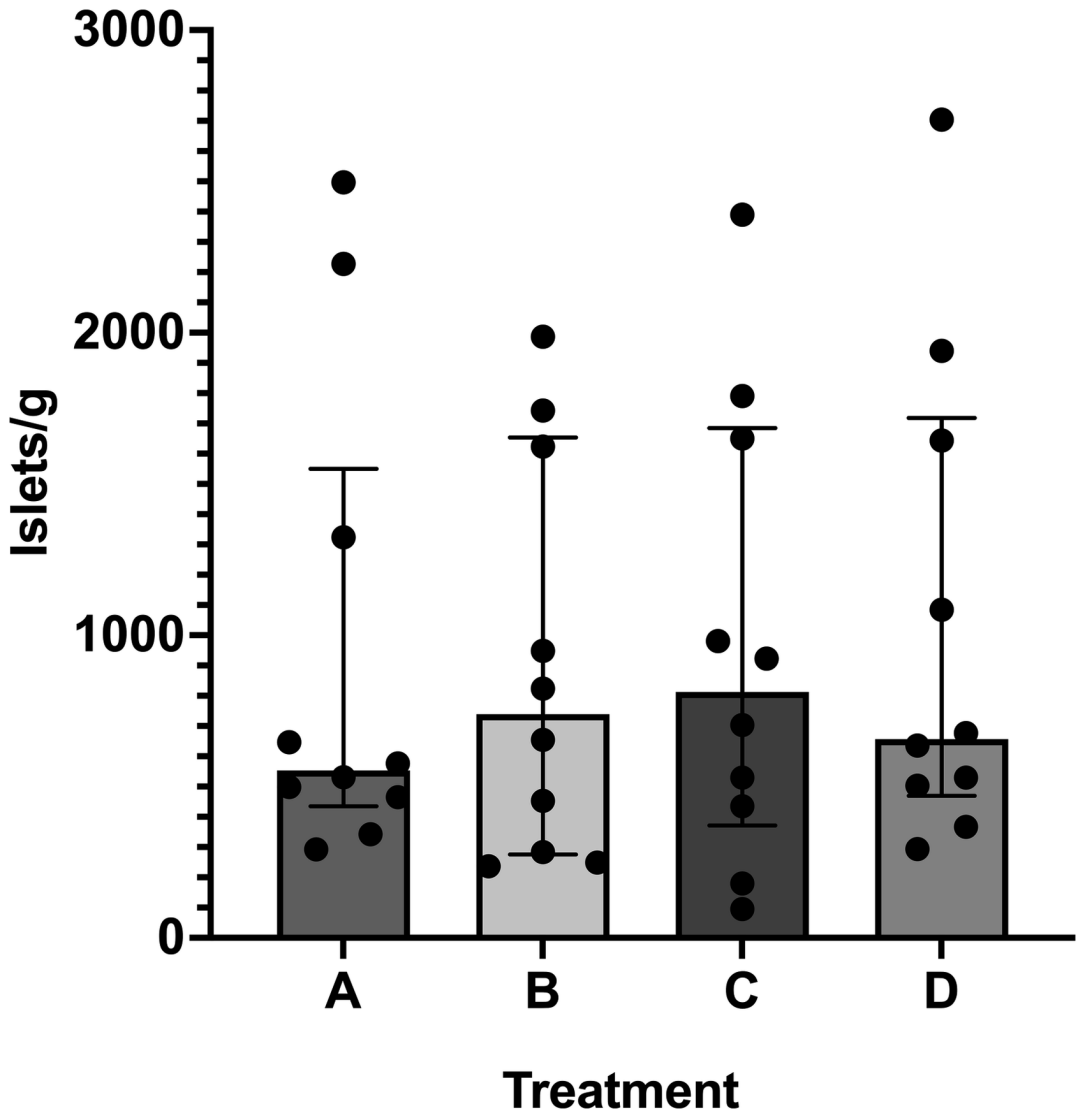
Islet yield among treatments. Histogram (median) and interquartile range (25 and 75 percentiles) depicting islet yield in treatment groups A (20 min incubation, 300 mmol/L glucose), B (40 min incubation, 300 mmol/L glucose), C (20 min incubation, 600 mmol/L glucose), and D (40 min incubation, 600 mmol/L glucose). For each treatment, *n* = 10. There was no difference in islet yield between treatment groups.

SOS produced isolated islets in all cats. Overall median (range) of islet yield was 690 (95–2,704) islets/g (*n* = 10). Treatment group did not have an effect on islet yield (*p* = 0.7) (Figure 4). Two outliers were identified in treatment group A, but even with removal of these outliers, there was no difference between treatment groups (*p* = 0.7). When evaluating the specific effects of osmolality of the glucose solution, islet/g median (range) for tissue exposed to 300 mmol/L glucose (611 [237–2,497], treatment groups A and B) was not different for tissue exposed to 600 mmol/L glucose (690 [95–2,704], treatment groups C and D). There was also no difference between exposure time on islet yield, in which tissue exposed for 20 min yielded 611 islets/g (95–2,497) and tissue exposed for 40 min yielded 666 islets/g (237–2,704). Islet yield differed among individual cats (*p* = 0.0025, Figure 1).

### Islet purity and morphology

**Figure 5:**
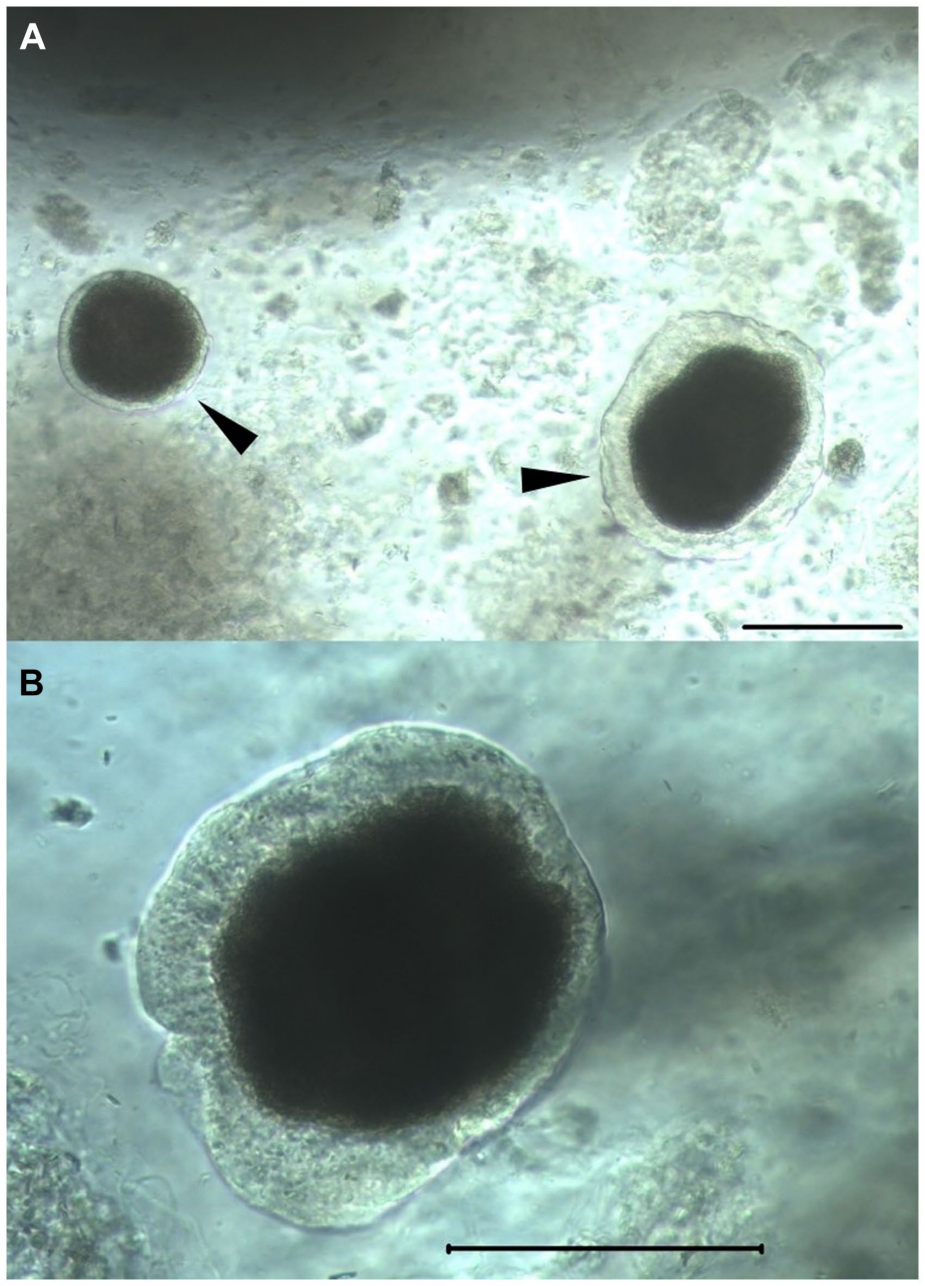
Isolated feline islets. Light microscopy photographs of feline islets isolated by SOS. Arrowheads **(A)** highlighting islets free of acinar cells. The surrounding floating amorphous material are acinar cells. Scale bars in both in panels **(A, B)**: 100 μm. Note the intact peri-insular capsule, a marker of islet health.

Islet purity (percentage of islets vs. acinar tissue) was estimated to be between 20 and 30% in all individuals and treatment groups, and there was no quantifiable difference between treatments. Visual assessment of morphology with light microscopy revealed islet sizes ranging from 50 to 150 μm. Observed islets were spherical, compact, and had well-rounded borders with congruent translucent capsular integrity (Figure 5). Subjectively, no differences in morphology were observed between treatments.

### GSIS

**Figure 6:**
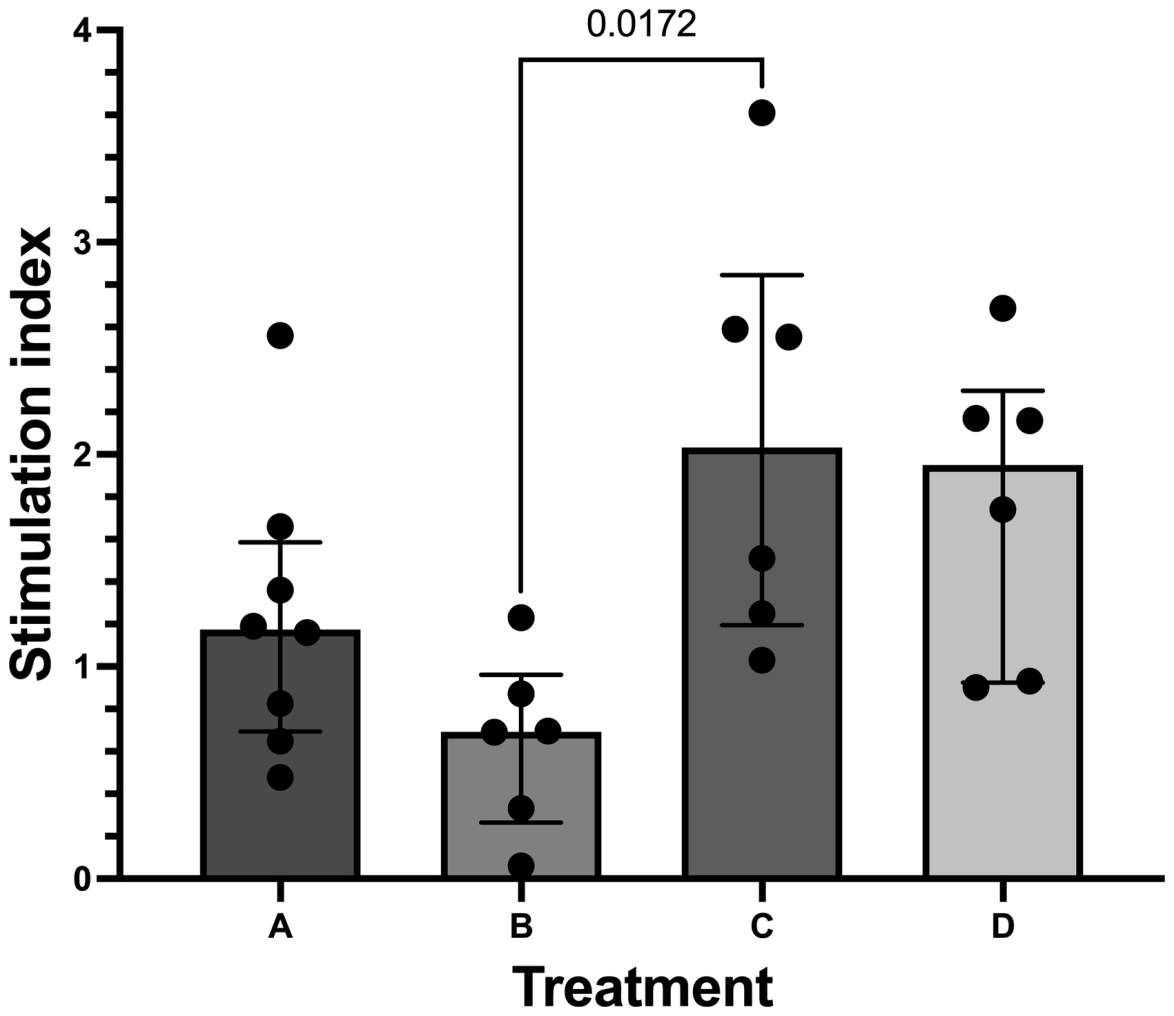
Stimulation index from glucose stimulated insulin secretion (GSIS). Histogram (median) and interquartile range (25 and 75 percentiles) depicting stimulation index (SI) in treatment groups A (20 min incubation, 300 mmol/L glucose) *n* = 8, B (40 min incubation, 300 mmol/L glucose) *n* = 6, C (20 min incubation, 600 mmol/L glucose) *n* = 6, and D (40 min incubation, 600 mmol/L glucose) *n* = 6. SI was significantly higher in treatment C compared to treatment B.

Median (range) stimulation index (SI) for all islets in the study that underwent GSIS was 1.19 (0.06–3.61). Treatment group C (600 mmol/L glucose for 20 min) was found to have a higher (*p* = 0.017) median SI (2.03 [1.19–3.61]) compared to treatment group B (300 mmol/L glucose for 40 min) (0.69 [0.06–1.23] Figure 6). When osmolality was considered, median SI was higher (*p* = 0.0032) in islets incubated at 600 mmol/L glucose (1.95 [0.902–3.61]) compared to islets incubated at 300 mmol/L glucose (0.85 [0.06–2.56]). No difference (*p* = 0.25) was found between 20-min incubation (1.3 [0.48–3.61]) and 40-min incubation (0.92 [0.06–2.69]).

## Discussion

To our knowledge, this is the first description of SOS islet isolation in cats, and our results show that the SOS isolation protocol produces glucose-responsive β-cells, supporting our hypothesis. GSIS is a well-accepted measure of islet function, and healthy islets are expected to secrete increased insulin concentrations when moved from low to high concentrations of glucose (21, 35). In other species, healthy islets typically produce stimulation index >2 (35–37), but the standard for SI in human islet transplantation is an SI >1 (21). This is because clinical outcome of human islet transplant is not correlated with SI (18). Stimulation index can also be variable due to differences in protocols between institutions (37) and potentially because of intrinsic differences between species (35–37). There is only one study that has published on GSIS in isolated feline islets, and although this study used a different incubation time (30 min) and high glucose concentration (16.7 mM), the mean SI was approximately 2 (32), which is similar to our results in treatment group C. *In vivo* insulin stimulation testing suggests that glucose is a less effective stimulator of insulin secretion in cats compared to humans and that arginine may be superior to glucose for assessing insulin secretory capacity (38). Our results showed that islets isolated with 600 mmol/L glucose solution had higher SI than islets isolated with 300 mmol/L glucose solution. In canine islets, no significant difference was found between these two osmolalities, however, islets isolated with 300 mmol/L glucose solutions had higher trending SI than those isolated with 600 mmol/L glucose (27). It was suggested that higher glucose concentrations in dogs may affect islet function and viability, and although no difference in islet viability was found between osmolalites, there was a higher percentage of acinar cell death in the higher osmolality treatments (26). Unlike dogs, our results suggest that a higher osmolality could be optimal for isolation by SOS in cats. However, viability was not assessed, which is one of the limitations of this study.

Feline islets may be more sensitive to collagenases than islets of other species, and one proposed reason for this is that there is less basement membrane-like material and collagen surrounding feline islets and separating them from acinar tissue (30, 31). Higher amounts of collagen directly surrounding islets has been associated with higher purity (39) after enzymatic islet isolation procedures in other species, while higher amounts of total collagen in the pancreas are also associated with more difficult islet isolation. Thus, it is possible that traditional enzymatic islet isolation methods have a causal relationship with the limited success in producing glucose-responsive feline islets in the literature. The use of collagenases inherently cause damage to islets, injuring basement membranes and extracellular matrix, which causes a localized pro-inflammatory response via free radicals and cytokines (23–25, 40). Islets have even been shown to internalize these enzymes, resulting in decreased function and cell death (24). This results in the selection for resilience to collagenase-based procedures, which is unlikely to be uniform among islet size, functionality, and micro-architecture (41–43). Conversely, SOS likely selects for islets that have a higher density of GLUT 2 transporters, which has been identified as a favorable indicator of islet function and glucose responsiveness, (26, 44, 45) desirable traits for transplantation as well as *in vitro* studies.

The SOS isolation method has been shown to yield higher islets per gram of tissue (13,423 islets/g) (26) compared to Liberase-treated porcine pancreata (4,210 islets/g) (46) and compared to human cadaveric pancreata (2,279 islets/g) (19). In dogs, the SOS protocol had lower yield (28×10^3^ islet-like equivalents) compared to traditional methods (49 to 234×10^3^ islet-like equivalents) (47), however, delay in quantification and warm ischemia are suggested to have affected yield (27). There is minimal information on feline islet yield, with a single publication showing a mean yield of islet-like cell clusters (±SD) to be 2,200 ± 1,400 from a mean 3.8 ± 1.0 g of pancreas (30). Other publications have not reported yield(29, 31, 32). SOS isolation in feline donors, however, seems to have similar to increased yield when compared to traditional methods. Warm ischemia during islet isolation could have affected islet yield, and performing this step under cooled conditions is recommended in the future. Our treatment groups were the same as in other studies on SOS, and we found that neither osmolality nor incubation time had an effect on islet yield. Thompson et al. had a similar finding in canine donors (27), while Atwater et al. (26) found highest yield in 600 mmol/L incubated for 20 min in porcine donors. It has been proposed that higher osmolality could increase yield while longer incubation time may increase damage to islets. However, our findings did not support this outcome in cats. Additionally, there was marked variability in yield among individual cats, despite being free of known pancreatic and circulatory disease, which could also affect our ability to perceive treatment differences. The explanation for such wide ranges in islet yields is unclear, but some potential explanations could include varying degrees of warm ischemia after harvest and subclinical pancreatic, cardiovascular, or metabolic disease that may have affected islet concentration. Unfortunately, histopathology was not performed to assess and compare pancreatic architecture and islet concentration differences among individuals, which is a limitation to this study. There was significant variability in human islet yield until Ricordi described a method for a semi-automation of the enzymatic isolation process (15, 19). It is possible that a similar system for automation of the degree of mechanical and chemical exposure of the islets will be required before the selective osmotic shock procedure results in consistent islet yield, as well.

There are no standard morphological assessment criteria for isolated islets, and morphology differs among species (48). Morphology scoring systems proposed in human islet xenotransplantation have shown to correlate with outcome, where higher scores consist of spherical islets, well-rounded borders, compact cells, and larger borders (49, 50). Peri-insular capsule integrity has also been recognized as marker of islet health, and in recent years collagenase-based methods have aimed to preserved to aim capsular integrity (25, 26, 46, 51, 52). Feline islets isolated by SOS meet the majority of high-quality morphological characteristics described in isolated islets of other species, with a notably prominent and congruent peri-insular capsule that has not been described or demonstrated in feline islets isolated by other methods (29–31).

SOS is technically simple, allows for the successful isolation of clinical grade quality islets from feline donors, and is considerably less costly than traditional methods. While this protocol will be useful in obtaining feline islets for *in vitro* studies of feline islet physiology, it still requires significant improvements in islet purity and yield before clinical application of islet transplantation. Following SOS, Thompson et al. (27) incorporated the use of a sieve and cell strainer prior to plating canine islets for cell culture. We found that, in feline islet isolation, this drastically compromised yield, therefore the only purification step performed was hand picking islets to separate them from acinar tissue. Visual estimation of purity is also a limitation of this study, and in the future the use of an image analysis software for quantification of purity is suggested. Clinical islet transplant materials for humans have significantly higher purity (78.5%), (50, 53) but collagenase-based techniques require an additional step in purity, such density-gradient centrifugation, which separates acinar tissue from islets using dextran or ficoll (19, 20). An additional step to improve purity and further refinement and automation of mechanical tissue disruption is likely needed for the current SOS protocol. Nevertheless, this protocol enables access to an additional islet model for human type II diabetes mellitus.

## Data availability statement

The raw data supporting the conclusions of this article will be made available by the authors, without undue reservation.

## Ethics statement

The animal study was approved by the Institutional Animal Care and Use Committee at the University of Florida. The study was conducted in accordance with the local legislation and institutional requirements.

## Author contributions

LP: Conceptualization, Data curation, Formal analysis, Funding acquisition, Investigation, Methodology, Project administration, Resources, Writing – original draft, Writing – review & editing. CA: Conceptualization, Investigation, Methodology, Project administration, Resources, Supervision, Writing – original draft, Writing – review & editing. CC: Data curation, Project administration, Writing – original draft, Writing – review & editing. JM: Data curation, Project administration, Writing – original draft, Writing – review & editing. CG: Conceptualization, Data curation, Formal analysis, Funding acquisition, Investigation, Methodology, Project administration, Resources, Supervision, Writing – original draft, Writing – review & editing.

## Footnotes

^1^https://www.protocols.io/view/potency-test-glucose-stimulated-insulin-release-as-36wgq5odkgk5/v3?step=49
